# Beyond Access: Telehealth Readiness, Trust, and Early Use Among Jordanian Patients with Chronic Illness

**DOI:** 10.3390/healthcare14091118

**Published:** 2026-04-22

**Authors:** Ahmad Rajeh Saifan, Murad Sawalha, Ibtisam A. Alarabyat, Hanan F. Alharbi, Zyad Saleh, Osama Alkouri, Rani Shatnawi, Dana Anwer Abujaber, Rami Eid Samarah, Nabeel Al-Yateem

**Affiliations:** 1 Faculty of Nursing, Yarmouk University, P.O. Box 566, Irbid 21163, Jordan; ahmad.saifan@yu.edu.jo; 2 Department of Maternal, Child and Family Health Nursing, Faculty of Nursing, The Hashemite University, Zarqa 13133, Jordan; msawalha@hu.edu.jo; 3 College of Nursing, Middle East University, Amman 11831, Jordan; ebtisamarabyat@gmail.com; 4 Maternity and Pediatric Nursing Department, College of Nursing, Princess Nourah bint Abdulrahman University, P.O. Box 84428, Riyadh 11671, Saudi Arabia; 5 Department of Clinical Nursing, School of Nursing, The University of Jordan, Amman 11942, Jordan; zyad.saleh@ju.edu.jo; 6 Department of Nursing, Vision College, Riyadh 11691, Saudi Arabia; 7 Basic Nursing Department, Nursing College, Yarmouk University, Irbid 21163, Jordan; o.alkouri@yu.edu.jo; 8 Respiratory Therapy Department of Allied Medical Sciences, Faculty of Applied Medical Sciences, Jordan University of Science and Technology, Irbid 22110, Jordan; rashatnawi@just.edu.jo; 9 Clinical Nursing Department, Faculty of Nursing, Applied Science Private University, Amman 11931, Jordan; d_abujaber@asu.edu.jo; 10 Nursing Department, Fatima College of Health Sciences, Abu Dhabi P.O. Box 3798, United Arab Emirates; sar_samara@hotmail.com; 11 Department of Nursing, College of Health Sciences, University of Sharjah, Sharjah P.O. Box 27272, United Arab Emirates; nalyateem@sharjah.ac.ae

**Keywords:** telemedicine, chronic disease, patient participation, patient acceptance of health care, qualitative research, Jordan, caregivers

## Abstract

**Background:** Telehealth has expanded access to care for people with chronic diseases, but little is known about how patients in Jordan become activated, motivated, and ready to use these services, particularly during early adoption. **Aim**: To explore how patients with chronic diseases in Jordan describe their initial activation, readiness, and experiences with telehealth services. **Methods:** This exploratory qualitative study used interviews with 14 purposively selected adults with chronic diseases from three hospitals in Jordan. Data was analyzed using Braun and Clarke’s six-step thematic analysis. **Results:** Four interrelated themes emerged. First, patients valued telehealth for preserving independence and ensuring continuity of care, particularly by reducing reliance on family members for transportation to health facilities. Second, readiness was shaped by geography, mobility, and finances. Although telehealth reduced transport costs and lost wages, patients still had to pay for devices and internet access, creating an economic paradox for poorer patients. Third, participation was supported by families but hindered by low digital literacy, platform changes, and unstable internet connectivity. Fourth, trust in telehealth was conditional and depended on patients’ perceptions of convenience and responsiveness. **Conclusions:** Readiness to use telehealth was relational, structural, experiential, and conditional rather than purely individual. Patients with chronic diseases in Jordan need hybrid care models that engage families and leverage affordable digital technologies to support sustained telehealth use for disease management.

## 1. Introduction

Telehealth has become an important approach to healthcare delivery, enabling patients to access services remotely via digital communication technologies. Telehealth has made it easier for many different groups of people to access healthcare services. The use of telehealth has made it easier for people living in rural or hard-to-reach locations to receive medical care through virtual consultations without having to travel very far to see their doctor [1]. In addition, telehealth has the added benefit of decreasing ancillary costs associated with healthcare, such as travel time and time taken off work [2]. With the introduction of real-time video consultation services, the speed at which patients receive care has improved, and the consistency of the care provided has increased. In particular, ref. [3] points out that real-time video consultations are one way telehealth services improve healthcare providers’ ability to engage in real-time clinical decision-making. Similarly, ref. [4] describes how technological advancements improve ongoing patient monitoring and support follow-up care for patients with chronic illnesses or conditions.

The COVID-19 pandemic has been a major catalyst for the expansion of telehealth and has highlighted both the benefits and the challenges of its adoption. For example, ref. [5] describes the significant increase in the number of telehealth services offered by hospitals and private practices during the pandemic, ref. [6] investigates whether telehealth has allowed patients and their providers to maintain continuity of care during the crisis, and ref. [7] raises questions about whether telehealth reduces total healthcare costs, given that telehealth services are being utilized more than before the pandemic.

A growing body of evidence shows that telehealth can support chronic disease management by reducing hospital readmissions [8], improving self-management [9], and enhancing clinical outcomes [8]. However, these benefits depend heavily on the engagement of key stakeholders, particularly patients and healthcare providers. Their attitudes, confidence, and health literacy influence whether telehealth is implemented successfully [10,11]. Patient activation, including the knowledge, skills, and confidence needed to manage one’s health and engage with telehealth, has been linked to better outcomes among people with chronic illnesses and therefore warrants specific attention [9,12].

Previous research has identified personal, sociocultural, and technological factors that shape telehealth adoption. Ease of use and digital literacy are especially important, as older adults and patients with limited technical skills often experience telehealth as difficult or burdensome [13]. Perceived usefulness also matters, as patients are more likely to adopt telehealth when they believe it improves communication, reduces costs, or supports self-management [9]. Other influential factors include socioeconomic status, education, urban–rural residence, and cultural preferences for face-to-face interaction [14,15].

Early experiences with telehealth show a mixed picture of facilitators and barriers. Patients often value telehealth for its convenience, reduced travel time, and support for follow-up care and medication management. User-friendly tools and confidence in self-monitoring can further strengthen patients’ sense of control [16]. At the same time, poor connectivity, technical problems, and usability challenges can reduce satisfaction and increase care burden, sometimes leading to disengagement [9]. Trust and perceptions of clinical adequacy are also important, as telehealth is often seen as suitable for routine care but less appropriate for complex assessments or physical examinations [15,16]. Emotional concerns, including loss of personal connection and privacy worries, may further affect early acceptance [17].

Healthcare providers and health systems also shape patient engagement with telehealth. Providers’ technical competence, confidence in remote assessment, and perceptions of workload can influence whether patients engage with telehealth early on [6,8]. At the organizational level, integration with electronic health records, technical support, and system interoperability can facilitate implementation, while administrative burden, limited equipment, and insufficient training can hinder it [18,19]. In resource-limited settings, poor broadband access, limited device availability, and restricted clinical capacity further constrain telehealth uptake [12,18].

Despite growing evidence, important gaps remain, particularly in Middle Eastern and other low- and middle-income settings. In Jordan, telehealth expanded rapidly during the last five years but remains underdeveloped in chronic disease management. Existing studies have focused mainly on emergency applications, specific disease groups, or provider-related barriers rather than the process of patient activation [8,14,18]. Quantitative studies suggest a gap between willingness to try telehealth and sustained use, but current evidence does not adequately explain this difference. More broadly, the literature has emphasized utilization and satisfaction while giving less attention to patients’ first experiences, including motivation, readiness, sociocultural negotiation, and trust, which may be critical to long-term engagement [6,20]. This research applies the Technology Acceptance Model [21], which recognizes the reasons why an individual decides to use a technology based on two factors: (i) perceived usefulness and (ii) perceived ease of use. The TAM is typically used in telehealth research; however, until this research, it has primarily been used to understand individual perceptions of telehealth use and has paid very little attention to the contextual or relational factors that influence an early adopter’s use of telehealth.

It is necessary to emphasize the importance of early telehealth adopters, as positive initial experiences significantly influence patients’ future use, motivation, trust, and desire to continue using telehealth services. Most previous telehealth research has focused primarily on overall utilization, satisfaction levels, and long-term outcomes. The literature has lacked examination of how patients first become willing to engage with telehealth and how their early experiences affect their willingness to continue using these services. A better understanding of this period of readiness is necessary to bridge the gap between patients who initially use telehealth and those who continue to use it, particularly in low- and middle-income countries.

The current study builds on existing qualitative research by examining patients’ readiness to engage with telehealth and their activation during initial engagement, rather than only identifying general barriers and facilitators. This study explores the multiple components of readiness (relational, structural, experiential, and conditional). It examines how factors such as family support, digital inequities, and trust impact patient engagement with telehealth in Jordan.

This study addresses this gap by qualitatively exploring the experiences of Jordanian patients with chronic diseases. It examines the personal, sociocultural, and technological factors that influence the decision to begin using telehealth, as well as the barriers and facilitators encountered during initial use, particularly regarding access, ease of use, and trust. It also explores patients’ views on the value of telehealth in meeting immediate health needs compared with face-to-face care. By focusing on Jordanian patients, this study aims to deepen understanding of the behavioral, emotional, and environmental factors shaping early telehealth adoption in chronic disease management and to inform the development of telehealth programs that are culturally appropriate, accessible, and trustworthy.

## 2. Materials and Methods

### 2.1. Study Design

This study employed an exploratory qualitative design to examine the experiences and perceptions of healthcare providers and patients regarding telehealth activation and engagement in chronic disease management in Jordan. This approach was appropriate given the complexity of the issue and the limited prior knowledge in this context [22]. The study was primarily informed by pragmatism, with interpretive elements to support understanding of participants’ experiences within their social and cultural contexts [23]. Researcher reflexivity was also considered in line with the Consolidated Criteria for Reporting Qualitative Research (COREQ) Domain 1.

### 2.2. Settings and Participants

The study’s subjects were recruited from three separate hospitals using a coordinated strategy. Clinical staff helped identify patients who met the inclusion criteria; once patients were determined to be eligible, the researcher contacted potential subjects directly to provide study information and obtain their consent. Through this method, researchers recruited subjects with relevant telehealth experience across many different healthcare settings. Firstly, the study took place at a large military referral hospital in the country. It offers a remote follow-up clinic service, also known as the electronic clinic service, whereby patients can request appointments and seek medical consultations via video conferencing on the Internet. Secondly, the study took place in a private hospital in Amman, known for its high healthcare standards and use of cutting-edge technology to deliver medical services to its clientele. It specializes in serving a wide range of clients, including Jordanians and expatriates. It offers telehealth services via the Internet, allowing patients to access healthcare from the comfort of their homes, including medical consultations and individualized treatment plans. Lastly, the study took place in a third private healthcare facility located in the Abdali district of Amman, the capital city of Jordan. It offers telehealth services to its clientele, allowing them to seek consultations by phone and video and to schedule remote follow-up appointments to monitor the progress of patients with chronic illnesses.

The research employed a purposive sampling approach, a recognized non-probability sampling method often used in qualitative research to select participants most likely to provide the most relevant and insightful data [24]. Patients were eligible for inclusion if they were 18 years of age or older, had a chronic disease diagnosis, spoke Arabic fluently, and had used telehealth services for at least 3 months. Patients were excluded for having no previous telehealth experience or for being unable to participate in interviews because of their medical condition (e.g., severe illness or cognitive impairment). As potential subjects were recruited with assistance from clinical staff, there was an opportunity for selection bias; specifically, subjects were likely more engaged with healthcare services or more willing to participate than patients whom clinical staff did not recruit. Nonetheless, researchers made significant efforts to recruit subjects from diverse backgrounds and experiences to obtain a broad range of perspectives.

Variation in age, condition type, and digital experience was considered during sampling to capture diverse perspectives. For qualitative studies, sampling is based on the concept of data saturation rather than numbers. Data saturation was reached at 14 patients because no additional themes emerged from subsequent interviews. This is in line with standard guidelines for conducting qualitative studies, which recommend using small but data-rich samples. Variations among the participants were observed in their ages, types of chronic diseases, the duration of their illness, and their engagement with telehealth services. While all participants had been familiar with telehealth services for at least 3 months, they were not equally adept at using technology.

### 2.3. Data Collection

For this study, the main data collection method employed is semi-structured interviews, a widely accepted approach in qualitative research that enables the collection of data on the depth and richness of participants’ experiences [25]. It is a balance of a set of predetermined questions, ensuring a more in-depth exploration of data in response to participants’ experiences [22]. It should be noted that the interview guide was carefully designed to address a series of key research questions, experiences, perceptions, challenges, facilitators, and improvement strategies related to telehealth. It is also worth noting that a pilot interview test was conducted with two participants to ensure the appropriateness of interview questions. It consists of six structured sections: demographic questions; experiences and perceptions; the role and impact of telehealth; activation and engagement; facilitators and challenges; and improvement strategies (see Table 1).

A structured approach was followed when recruiting participants to avoid any bias in the study. This recruitment was conducted with the help of clinical staff, who were not involved in the actual recruitment process. Interviews were conducted in private rooms at any of the selected hospitals or at other locations, such as community centers, as preferred by the interviewees. Each interview lasted 45 to 90 min, depending on the flow. To ensure the accuracy and completeness of data collected in this study, all interviews were digitally recorded using a secure audio recorder after obtaining consent from the interviewees. In addition, field notes were taken during each interview, recording non-verbal cues such as body language and emotional tone.

### 2.4. Data Analysis

Data was analyzed systematically. The researcher manually transcribed interviews to support close engagement with the data [26]. As the interviews were conducted in Arabic, transcription and translation into English were completed simultaneously, guided by WHO recommendations [27]. Accuracy was supported through systematic back-translation. NVivo version 15 was used for data management. The analysis followed Braun and Clarke’s [26]. Six-step thematic analysis: familiarization with the data, line-by-line coding, generation of themes, review of themes, definition and naming of themes, and report writing. The coding process was conducted by the main researcher and independently verified by another team member to improve analytical quality. Codes were initially produced from the data set using inductive coding techniques applied line by line. Meetings were consistently held by the researchers to cross-check the coding processes and improve them where necessary. Themes were produced collectively through an iterative process of improvement.

The use of thematic analysis as a formalized form of qualitative data gathering provides a systematic yet flexible means of identifying, interpreting, and analyzing patterns in qualitative data. Thematic analysis was therefore deemed particularly appropriate for exploring patients’ perceptions and interpretations of their experiences with telehealth. This enabled the capture of both overt and covert themes in qualitative data on the use of telehealth.

### 2.5. Trustworthiness and Ethics

Ethical procedures were followed throughout the study. Before the commencement of the research, informed consent was obtained from the participants after they were briefed on the purpose, procedures, potential harms, and benefits of the research. The participants were informed that participation was voluntary and that they could withdraw at any time without consequences. Participants’ information was anonymized, and the data collected was stored securely on password-protected devices, with access limited to the research team.

To ensure the trustworthiness of the findings, the research adopted a number of strategies. To ensure credibility, the research used member checking to verify that the findings accurately reflected the participants’ experiences [28]. To ensure transferability, the research provided a thick description of the research context to allow the reader to judge the research’s applicability to their own context [29]. To ensure dependability, the research included an audit trail to record the entire research process. To ensure conformability, the researcher maintained a reflexive journal to record thoughts that could affect the research [29]. These strategies ensured the research conformed to the requirements of the COREQ Domain 1 by ensuring the research’s reflexivity.

The research team consisted of nurses (IA, HA, DA, and RS) who had used telehealth, qualitative health services researchers (AS, MS, and NA), and three research assistants (RS, OK, and ZS) who had some experience with the local health care system. Many of the team members had previous clinical experience with similar patient groups and believed that telehealth would facilitate access; we acknowledge the potential for bias from these viewpoints in framing questions, probing for clarification, and interpreting responses. We used several strategies to minimize potential bias: (1) interviewer reflexivity, whereby each interviewer created a reflexive fieldnote following each interview detailing their initial thoughts on the interview; (2) researcher triangulation, whereby two researchers coded transcripts independently, and compared their coding, discussing any inconsistencies with a third team member to resolve differences; (3) an audit trail by maintaining detailed records of the decisions made about coding (codifying), the creation of the codebook, and the analytic memos; (4) analytic reflexive meetings where all members of the research team regularly met to review how their backgrounds may impact the development of themes; and (5) participant validation, whereby we sought feedback from the participants regarding whether our interpretations made sense; if we were able to do so, we provided a summary of key findings to a member of the patient advisory group for verification. These practices allowed for increased accountability and credibility of the analysis, while stating that all interpretations are contextually located.

## 3. Results

This section presents an exploratory qualitative analysis of Jordanian patients’ experiences of initial activation, readiness, and use of telehealth for chronic disease management. Telehealth was not experienced simply as a substitute for face-to-face care, but as a different mode of managing healthcare shaped by independence, social support, affordability, technological capacity, and trust.

### 3.1. Participant Characteristics

The study included 14 patients recruited from three major healthcare organizations in Jordan: King Hussein Medical City, Abdali Hospital, and Specialty Hospital. Participants were aged 36 to 73 years, had education levels ranging from high school diplomas to bachelor’s degrees, and were living with chronic conditions including diabetes, hypertension, cardiac disease, and cancer. Disease duration ranged from 2 to 10 years. All participants had used telehealth through videoconferencing platforms (Table 2).

### 3.2. Thematic Analysis Overview

The analysis showed that patients understood telehealth through the realities of chronic illness, family life, and everyday care rather than as a purely technical intervention. Readiness was not described as an individual attribute, but as something negotiated through patients’ desire to remain connected to care, their social and material resources, and their actual experiences with telehealth. A summary of the themes, subthemes, and illustrative quotations is presented in Table 3.

Theme 1: Maintaining independence through connected care

For most participants (12 of 14), telehealth became meaningful not because of interest in technology itself, but because it helped them maintain continuity of care while reducing dependence on others. Many described telehealth as preserving autonomy, dignity, and self-respect by reducing reliance on family members for transportation and logistics.

As one elderly cardiac patient explained:

*“At my age, depending on others for transportation to appointments made me feel helpless. Telehealth restored my independence. I can manage my heart condition without being a burden to my family, which motivates me to stay engaged with the technology despite the learning effort.”*

(P1)

For some participants, telehealth also encourages a more active role in care. Rather than remaining passive recipients of advice, they described becoming more informed, observant, and confident in their interactions with healthcare providers.

One participant with hyperthyroidism stated:

*“Telehealth forced me to become more educated about my conditions. I learned to track my symptoms, understand my lab results, and ask better questions. This knowledge makes me feel like a partner in my care rather than a passive recipient, which keeps me motivated.”*

(P2)

Others valued telehealth because it maintained reassurance and continuity through regular communication with providers. A participant with diabetes described telehealth as a “game-changer,” noting that it enabled discussion of blood sugar levels, medication adjustments, and feeling “just as cared for” as during in-clinic visits (P9).

However, not all participants found telehealth empowering. Two participants described their use of telehealth as more obligatory than motivating, especially because they missed the emotional and relational aspects of in-person care. One participant noted:

*“I continue because I have to, but I’m not motivated by choice. I miss the personal touch of traditional appointments.”*

(P4)

Overall, activation was strongest when telehealth supported independence, continuity, competence, and connection, and weaker when it was experienced as a compromise to the relational value of face-to-face care.

Theme 2: Structural and financial shaping of telehealth readiness

Readiness to begin and sustain telehealth was shaped not only by motivation, but also by structural conditions such as geography, physical mobility, and financial resources. Telehealth lowered the barrier to accessing care, particularly for patients who faced long travel distances or physical strain in attending healthcare facilities.

One participant explained:

*“Living two hours from the nearest specialist meant I often skipped follow-up appointments. Now I can see my endocrinologist and cardiologist regularly without the exhausting journey.”*

(P2)

Similarly, a participant with mobility limitations described how telehealth reduced the physical burden of care:

*“Having mobility issues makes medical appointments increasingly challenging. Connecting with my rheumatologist from home means I don’t worry about transportation, stairs, or uncomfortable waiting room chairs.”*

(P14)

Telehealth was also described as economically beneficial because it reduced indirect costs such as transport, parking, and lost wages. For some, these savings improved their ability to afford medications. One participant stated:

*“Between transportation costs, lost wages from time off work, and parking fees… each specialist visit used to cost me nearly a day’s income… The financial relief has allowed me to afford all my medications consistently.”*

(P7)

At the same time, four participants described telehealth as financially burdensome because of the cost of smartphones, devices, or internet access. One participant noted:

*“For someone on disability income, these extra costs strain our already tight budget.”*

(P13)

These accounts show that readiness was materially constituted. Telehealth could reduce traditional barriers to care, but it could also reproduce inequality when patients lack the resources needed for digital access.

The participants were aware that their digital skills were inadequate and that Internet services were unstable, which are part of a wider set of challenges associated with the healthcare sector in Jordan. Many participants mentioned that the lack of Internet connectivity in rural areas and the cost of data and devices make using telehealth challenging, thereby maintaining existing inequalities rather than helping to reduce them.

Theme 3: Support networks and technological fragility in participation

Patients’ readiness to participate in telehealth was both relational and technological. Family support was a major facilitator for 11 of the 14 participants. Family members often helped with downloading applications, troubleshooting technical problems, and being present during consultations. In this sense, telehealth was often a shared family practice rather than an individual activity.

One participant explained:

*“My husband’s involvement made all the difference. He learned the technology alongside me… This shared responsibility transformed telehealth from a burden into a manageable part of our routine.”*

(P9)

However, reliance on family support complicated the idea that telehealth necessarily promoted autonomy. For some, telehealth reduced dependence in one area while creating dependence in another. As one participant stated:

*“My grandson helps me with video calls, and without him, I’d be lost… I worry something will go wrong or I’d disconnect during important conversations.”*

(P1)

Usability was also uneven. While some participants found the platform intuitive, others struggled because of age, physical limitations, or repeated platform updates. A participant with arthritis described the effect of interface changes:

*“The constant platform updates mean relearning the interface repeatedly. Buttons move… For someone my age with arthritis affecting my hands, these changes create significant barriers to accessing care.”*

(P11)

Technical problems further undermined participation. Six participants reported that poor internet connectivity or interruptions in video and audio affected not only convenience, but also communication, reassurance, and psychological comfort during consultations. One participant explained:

*“When the video stops during discussions about treatment, there is tension… These technical obstacles directly affected my psychology.”*

(P5)

This theme shows that telehealth participation depended on the alignment of family support, bodily capacity, stable design, and reliable technology. When these conditions were disrupted, readiness became fragile.

We noted two specific trends regarding family involvement. The first trend involved facilitating autonomy by supporting family members in technology use and engagement, which led to greater empowerment among patients. For instance, participants who received instruction from family members felt more empowered (e.g., P9). The second trend involved dependency, which manifested itself through the lack of autonomy or the emergence of vulnerability due to dependency, as shown by participants who described themselves as “lost” when there was no help from their relatives (e.g., P1).

Theme 4: Conditional trust in digital care

Patients did not view telehealth as inherently trustworthy or untrustworthy. Rather, trust was negotiated according to the type of encounter, the seriousness of symptoms, and whether telehealth could provide what patients believed proper care should involve.

Most participants did not describe digital care as emotionally distant. Ten of the 14 participants reported effective communication and felt that trust could still be built through attentive and responsive interaction. One participant stated:

*“What impressed me most was how caring and attentive my doctor and nurse were to me, even though it was through a computer screen. They take real time to answer questions… making me feel valued.”*

(P14)

Some also found that digital tools improved understanding. A participant explained:

*“Visual communication tools like screen-sharing test results helped me understand my condition better than verbal explanations.”*

(P12)

However, trust had clear limits. Participants expressed uncertainty when telehealth required them to interpret symptoms without physical examination or direct clinical observation, especially in complex or worsening conditions. One participant said:

*“I worry constantly about whether I’m monitoring the right things. When I feel short of breath… Without a doctor there to check, I sometimes panic unnecessarily.”*

(P8)

Another participant highlighted the limits of telehealth in expressing pain and being physically assessed:

*“When my pain levels change… it is hard to tell what I feel exactly without seeing the doctor personally.” *

(P5)

These accounts suggest that telehealth was viewed as appropriate for follow-up, medication management, reviewing results, and maintaining continuity, but less appropriate for complex cases, worsening symptoms, or situations requiring physical examination. Trust was therefore conditional and situational.

### 3.3. Integrative Interpretation of the Findings

Across all themes, readiness for telehealth emerged as a fluid social process rather than an individual decision to use technology. Patients engaged with telehealth when it helped them maintain independence, continuity of care, and access to care while reducing travel and practical burdens. However, this engagement depended on family support, financial resources, technological stability, and confidence that telehealth was appropriate for the clinical situation.

The findings suggest that telehealth readiness should not be reduced to digital literacy or patient motivation alone. It is relational because family supports it; structural because it is shaped by geography and finances; experiential because it is influenced by actual use; and conditional because it depends on trust in telehealth’s ability to meet the demands of a particular encounter. Telehealth was therefore not simply a replacement for conventional care, but a negotiated form of care shaped by both its possibilities and its limits.

## 4. Discussion

This study provides an in-depth qualitative account of how Jordanian patients with chronic diseases experienced initial activation, readiness, and use of telehealth. Telehealth emerged as a negotiated form of care that reshapes everyday illness management—supporting independence and continuity for some while introducing new dependencies and inequities for others. Readiness was not a fixed individual attribute; it was relational and continually reassessed. Patients’ use of telehealth was influenced not only by convenience but also by its ability to support independence, reduce burden, maintain continuity of care, and match clinical needs. At the same time, telehealth depended on family support, digital resources, reliable technology, and perceptions of care adequacy. Furthermore, the results confirm the TAM. Improved access to services and continuity of care were demonstrated as evidence of usefulness. Similarly, the ease with which the individual can use telehealth was influenced by their digital literacy and the technology problems they have experienced [30]. However, this research extends the TAM by demonstrating that an individual’s readiness to use telehealth is also based on relational, structural, and conditional factors, including family support, financial resources, and situational trust.

A major finding was that telehealth helped patients maintain independence while staying connected to care. Patients valued telehealth not only because it improved access, but also because it reduced reliance on others for transport and logistics. In this sense, telehealth had both practical and moral value, especially for older adults who did not want to feel like a burden to their families. This aligns with the literature linking telehealth to dignity and social personhood [4] and underscores household-level drivers of activation.

Activation was interpretive: telehealth helped some patients become more informed and prepared, repositioning them from passive recipients to active participants [20]. At the same time, not all patients were motivated by telehealth, indicating that telehealth use should not be equated with telehealth acceptance.

Place, finances, and day-to-day capacity also shaped readiness for telehealth. Telehealth improved access for rural patients and those with mobility limitations by reducing travel requirements and making care more physically manageable. These findings support evidence that telehealth can reduce geographic and logistical barriers to care [1,2] and lower travel-related costs in Jordan and Saudi Arabia [18,31]. To place barriers to connectivity and digital literacy in their context, we look at Jordan’s relative availability of broadband, device/data costs, and the discrepancy in digital health investment; these also shape how telehealth can transform barriers to access, rather than remove them.

However, this study complicates the assumption that telehealth is inherently accessible or affordable. Although telehealth reduced travel costs, parking fees, lost wages, and physical strain, it introduced new demands, including devices, internet access, mobile data, and digital skills. Telehealth, therefore, shifted barriers rather than eliminating them. This extends Yoon, Hur [10] by showing how income- and literacy-related disparities also shape chronic care access in Jordan. Socioeconomic factors did not simply influence readiness; they produced it. In this case, it is necessary to consider the full set of factors within Jordan’s healthcare infrastructure, including broadband availability, device and data costs, and digital health investments. Therefore, telehealth preparedness should be considered at the system level, including efforts such as improving broadband availability, subsidizing devices and data for low-income patients, and incorporating telehealth into regular service provision.

One of the most distinctive contributions of this study is its identification of telehealth readiness as socially distributed rather than individually held. Family support was not merely an aid to participation, but part of participation itself. Many patients relied on spouses, children, or grandchildren to manage devices, platforms, and consultations. This extends previous work on digital literacy barriers by showing that apparent individual readiness may actually reflect household readiness [32].

This is especially important in the Jordanian context, where family interdependence has strong social and cultural significance. Much of the existing literature, particularly from Western settings, emphasizes individual usability and digital competence. In contrast, the present findings show that readiness is often socially shared. At the same time, this also reveals vulnerability, as patients without family support were more likely to experience anxiety and disengagement. In our consideration of family dynamics, we label two types of involvement, including supportive facilitation compared to dependence dynamics and note outcomes for design and training.

Relatedly, the study shows that telehealth does not remove dependency so much as reconfigure it. Patients may become less dependent on others for transportation while becoming more dependent on others for digital access. This challenges the assumption that telehealth straightforwardly promotes autonomy. It must be noted that family support can be viewed as a continuum between support and dependence, with the outcome depending on the methods of intervention (i.e., enhancing patients’ individual skills through user-centered design while empowering family members as sources of support, rather than creating a new form of dependency).

The findings further show that participation depended not only on support but also on technological reliability. Interface usability and internet stability affected not just access, but also trust and the therapeutic quality of the consultation. Patients did not experience these as minor technical inconveniences; they shaped the emotional tone of encounters, especially during discussions of treatment or symptom changes. This adds to the literature on user comfort and technology acceptance by showing that technological reliability is part of the therapeutic environment itself [33].

Trust in telehealth was also more complex than a simple contrast with face-to-face care. Patients described trust as conditional and negotiated according to what they believed a particular clinical encounter required. Many reported that clinicians were warm, attentive, and communicative during telehealth consultations, showing that emotional connection is possible through digital care. Some also found that tools such as screen sharing improved understanding and engagement. However, trust weakened when symptoms were difficult to interpret, when pain or breathlessness worsened, or when physical examination was important. This is consistent with earlier research showing that patients often prefer a combination of telehealth and in-person care, particularly for diagnostic complexity or urgent concerns [15,16]. The present study extends this literature by showing that such preferences are rooted not only in practical concerns but also in beliefs about what constitutes good care. Three different types of trust which are associated with the adoption of telehealth are described below: (1) Trust in technology, which is associated with ease of use, availability, and security; (2) Trust in clinicians, which is associated with how clinicians communicate and pay attention to patients; and (3) Trust in systems, which is associated with the accessibility, availability, and governance of telehealth services. These types of trust can influence each other, as technical issues can affect doctor–patient communication even when doctors do care.

Compared with studies from the region and internationally, the experiences of Jordanian patients in this study show both similarities and differences. As in studies from Saudi Arabia and Bahrain, telehealth uptake was shaped by sociodemographic and infrastructural conditions [14,33]. However, unlike some regional and international literature, participants in this study were more concerned with usability and clinical adequacy than with privacy. This suggests that for patients managing chronic illness, the central issue is not only whether telehealth is secure, but whether it is workable and clinically sufficient over time. In addition, whereas earlier studies emphasized telehealth as a response to COVID-19-related continuity needs [34]. Participants in this study viewed telehealth more as a long-term care modality than as a crisis measure.

These findings have practical implications. For nurses, telehealth readiness assessment should go beyond willingness and digital literacy to include family support, confidence in symptom monitoring, and whether telehealth suits the patient’s current clinical needs. For physicians, readiness should include clearer triage to determine when telehealth is appropriate and when a face-to-face assessment is needed. For healthcare managers, telehealth planning should consider family support and technological reliability because both affect trust and continuity of care. For policymakers, readiness should also include affordability, since device and connectivity costs may deepen disadvantage. Overall, the findings suggest that telehealth for chronic disease management should be designed as a hybrid model rather than a full substitute for face-to-face care.

Taken together, these findings show that telehealth readiness in chronic disease care should not be understood only as digital willingness or literacy. It is relational because family members often make participation possible; structural because it is shaped by geography, finances, and access to technology; experiential because it is strengthened or weakened through use; and conditional because trust depends on whether telehealth matches the needs of the clinical moment. Telehealth is therefore best understood not as a replacement for conventional care, but as a negotiated form of care shaped by both its possibilities and its limits.

### Strengths and Limitations

There are several limitations to this research. First, the research included only individuals who had been telehealth users for at least three months; those who had used telehealth for less than three months were not included in the study. According to the findings, participants may not have varied in the amount of contact they had with the healthcare provider, and there may have been selection bias toward individuals with longer histories of use who might have had a better experience. As a result, the study sample could not represent all individuals who used telehealth; individuals who decided not to use telehealth or discontinued their use are not represented in this study. Second, participants were recruited through three major healthcare systems, which may limit the generalizability and power of the results to smaller healthcare systems or rural healthcare settings, where the availability of healthcare technology, support, and providers may differ from those of the three major healthcare systems used to recruit this study’s participants. Third, this article focuses on patients’ experiences, so it is most directly about their experiences and understanding of telehealth; it does not provide perspectives from other individuals involved in the telehealth implementation process. In conclusion, while focusing on patients is a potential limitation, exploring patients’ experiences with telemedicine in a clinical context represents a substantial contribution to our understanding of how telehealth is used and how patients perceive it.

## 5. Conclusions and Implications

This study concludes that patient activation in Jordan is socially constructed and shaped by empowerment, economic trade-offs, and family infrastructure. Although readiness was higher among patients with support systems, those without such support may be at risk of exclusion. A hybrid telehealth model that incorporates scheduled in-person appointments for symptom evaluation or significant change, routine follow-up via virtual methods, and simple triage protocols and checklist-style documentation of telehealth readiness should be the norm. Policymakers should also ensure that patients have access to devices/data (through additional subsidies) and have access to sufficient broadband to use telehealth, while supporting health services to create user-friendly platforms and train healthcare providers on how to communicate effectively by video and properly escalate issues during these interactions (including how to engage family caregivers). Finally, health services should continually assess telehealth equity and outcomes and make ongoing improvements to the delivery of health services through telehealth. Future work should evaluate targeted interventions (for example, subsidized connectivity, family-inclusive training programs, and hybrid service models) in representative, longitudinal studies to assess their effectiveness, scalability, and equity of implementation.

## Figures and Tables

**Table 1:** Interview Guide Questions.

Opening Introduce researcher, purpose, confidentiality, recording, length (~45–60 min). Obtain consent. Warm-up: Can you briefly tell me about yourself (age, chronic condition(s), how long you’ve had it, and experience with telehealth)? Section A—Activation, motivation, readiness How did you first learn about telehealth services, and what made you decide to try it? What were your main reasons or motivations for starting telehealth? How ready did you feel when you first used telehealth? What made you feel ready or unready? Section B—Personal, socio-cultural, and technological influences 4. What personal factors (health, skills, beliefs) affected your decision to start telehealth? 5. How did family, friends, or community influence your decision to use telehealth? 6. What technological or infrastructural factors influenced your decision (device access, internet, platform availability)? Section C—First experiences: barriers and facilitators 7. Tell me about your first telehealth appointment. What went well and what went wrong? 8. Which factors helped you complete that first session successfully? 9. Which barriers did you face during early use, and how did they affect you? Section D—Access, usability, trust 10. How did telehealth change your ability to access care compared with in-person visits? 11. How easy or difficult do you find using the telehealth platform(s)? What specific features are helpful or problematic? 12. How much did you trust the care you received via telehealth? Why or why not? Section E—Role vs. face-to-face care and perceived outcomes 13. For what kinds of problems do you prefer telehealth vs. in-person visits? Can you give examples? 14. How has using telehealth affected your ability to manage your chronic condition (self-management, medication adherence, reassurance)? 15. Have there been any unintended consequences (positive or negative) from using telehealth? Section F—Sustainability and improvements 16. What would make you continue using telehealth regularly? What would make you stop? 17. If you could change one thing about how telehealth is provided, what would it be?

**Table 2:** Patient Participant Characteristics.

Participant	Age	Gender	Educational Level	Chronic Condition	Years with Condition
P1	73	Male	High School	Cardiac Disease	4
P2	65	Female	Bachelor’s	Hyperthyroidism, Diabetes, Hypertension	6
P3	73	Male	High School	Diabetes, Parkinson’s Disease	8
P4	67	Female	High School	Chronic Heart Disease, Hypertension	8
P5	36	Female	Diploma	Diabetes	3
P6	55	Female	High School	Diabetes & Bone Marrow Cancer	4
P7	62	Male	Bachelor’s	Gout, Atrial Fibrillation, Chronic Kidney Disease	9
P8	45	Female	Bachelor’s	Cardiomyopathy, Bronchial Asthma	4
P9	65	Male	Bachelor’s	Diabetes, Hypertension	10
P10	62	Female	Bachelor’s	UTI, Diabetes, Hypertension	7
P11	56	Female	Bachelor’s	Hypertension & Knee Joint Pain	6
P12	46	Male	Bachelor’s	Coronary Artery Disease	2
P13	58	Male	High School	Chronic Kidney Disease	5
P14	51	Female	Diploma	Diabetes &Rheumatoid Arthritis	4

**Table 3:** Summary of Themes, Subthemes, and Illustrative Quotations.

Theme	Description	Key Subthemes	Illustrative Quotes (Short)
Theme 1: Maintaining independence through connected care	Telehealth enabled patients to preserve autonomy while remaining engaged with healthcare services.	Autonomy, reduced reliance on caregivers, continuity of care	“Telehealth restored my independence…”
Theme 2: Structural and financial shaping of telehealth readiness	Readiness for telehealth was influenced by geographic access, mobility, and financial capacity.	Geography, transportation burden, financial cost and affordability	“Living two hours away…”
Theme 3: Support networks and technological fragility in participation	Participation depended on family assistance and was challenged by usability and technical instability.	Family support, usability challenges, and internet/connectivity issues	“My husband’s involvement…”
Theme 4: Conditional trust in digital care	Trust in telehealth was context-dependent and shaped by perceived clinical adequacy and communication quality.	Clinical adequacy, symptom uncertainty, and communication quality	“I worry about monitoring…”

## Data Availability

The data presented in this study are available on request from the corresponding author due to privacy and ethical restrictions.
